# Is there an added value in more real‐world cannabinoids studies?

**DOI:** 10.1002/ejp.2071

**Published:** 2022-12-28

**Authors:** Elon Eisenberg

**Affiliations:** ^1^ Pain Research Unit Institute of Pain Medicine, Rambam Health Care Campus Haifa Israel; ^2^ The Ruth and Bruce Rappaport Faculty of Medicine Technion ‐ Israel Institute of Technoology Haifa Israel

This journal recently published a paper by Horsted et al. (2022) entitled ‘Safety and Efficacy of Cannabinoids to Danish Patients with Treatment Refractory Chronic Pain—A Retrospective Observational Real‐world Study’. Eight hundred and twenty‐six patients with ‘refractory’ chronic pain, were prescribed oral cannabinoids consisting of either delta‐9‐tetrahydrocannabinol 14 (THC), cannabidiol (CBD) or their combination. Data on adverse effects, pain reduction, sleep quality and quality of life were collected roughly 2 and 4 months (median) following treatment initiation. Nearly two‐thirds of the patients were included in the first follow‐up analyses and only a quarter in the second. An intention‐to‐treat (ITT) analysis of 826 eligible patients revealed clinically relevant pain reduction (NRS ≥30%) in 17% and 10% at the two follow‐ups or in 32% and 45% of 529 and 214 analysed patients, respectively, according to a per‐protocol (PP) analysis. Per‐protocol analyses also revealed improvement in sleep quality and in quality of life in ~50%. Adverse events were reported by 42% and 34% of the patients at the two follow‐up time points and were mild to moderate in general.

Although the medicinal use of cannabinoids continues to expand, ongoing attempts fail to provide clear evidence of their effectiveness and safety for pain. A considerable number of randomized controlled trials (RCTs) and subsequent systematic reviews and meta‐analysis (SRMA) have not been translated into solid clinical evidence or practice guidance on cannabinoids for chronic pain (Eisenberg et al., 2022).

To increase the foundation of evidence in such circumstances, GRADE (Grading of Recommendations Assessment, Development and Evaluation) allows to consider real‐world observational studies if pointing to consistent and large effects. Indeed, several long‐term observational studies on cannabinoids in chronic pain have been published and six of them were even included in a recent SRMA (Bialas et al., 2022). It concluded that: ‘within the context of observational studies—which should be regarded with caution—cannabis‐based medications had positive effects on multiple symptoms for some chronic pain patients and were generally well tolerated and safe’.

The percentage of patients achieving 30% pain relief in the Horsted et al. (2022) study, which ranges between 32%–45% in the PP analysis and 10–17% in the ITT, seems congruent with previous reports. For example, Bialas et al. (2022) reported of 38.3% of patients achieving the 30% target in all six included studies, and 20.5% after removing four studies for which an imputation method was used to calculate responder rates. The same is probably true for the improvement in secondary outcomes such as sleep and quality‐of‐life, and for the rates and types of reported adverse events. Hence, the authors concluded that ‘oral cannabinoid therapy seems to be safe and mildly effective in patients with chronic pain’ (Horsted et al., 2022) may strengthen what is already known, but is marginal in terms of added value. An additional major drawback of this and most other real‐world observational studies is their low patient retention rate (26% in the second follow‐up in this study), which creates a significant bias and makes the results difficult to interpret. Hence, methods of improving patient compliance such as identifying and focusing on key outcomes only, adopting user‐friendly outcome recorders, standardizing data collection, etc., should be considered in future studies.

At the same time, the study does provide some valuable data. First, although the exact cannabinoid titration regimen is not specified in the study, it still shows that relatively low mean doses of THC (14 or less mg/day) and CBD (35 or less mg/day) are sufficient for producing the observed analgesic effect. These doses are lower than those used in previous studies. Second, the study design allowed a comparison between THC, CBD and their combination and indeed showed that patients prescribed THC/CBD were significantly more like to obtain a ≥ 30% pain reduction than THC and CBD as monotherapies. These two findings can clearly be helpful for planning treatment regimens in future RCTs.

We have learned that recruiting patients to analgesic RCTs based on their underlying pain conditions (i.e. neuropathic vs. nociceptive, or post‐herpetic neuralgia vs. painful diabetic neuropathy) is often insufficient to show efficacy, thus leading to frequent failure of phase II and III analgesic RCTs. However, stratifying patients into sub‐groups according to pain mechanism, patient‐reported outcomes, etc., helped to identify responding versus non‐responding patients and increased assay efficacy. The same principles can also be applicable in real‐world life studies. In other words, rather than looking at the outcomes of the entire group such as magnitude of pain reduction or effect size (which is often the case in observational studies), one should probably consider categorizing patients into ‘responders’ and ‘non‐responders’ based on a simple validated outcome, and then, based on a broad baseline assessment, identify predictors for becoming a ‘responder’. This will require a careful, multi‐modal and comprehensive baseline assessment of patients, but by focusing on specific outcomes only—will reduce follow‐up burden upon patients and enhance patient compliance.

In summary, the growing use of medicinal cannabinoids and the failure of RCTs to provide clear evidence for their efficacy and safety in patients with chronic pain justify the use of additional study designs such as Horsted et al. (2022) real‐world observational study. In line with previous real‐world studies, this study's results suggest a reasonable safety profile and modest effectiveness. However, methodologies enabling stratification of patients into subgroups (i.e. ‘responders’ vs. ‘non‐responders’) and enhanced patient retention rates will be required for obtaining significant added value in future real‐world studies.

## FUNDING INFORMATION

None.

## CONFLICT OF INTEREST

Elon Eisenberg received consulting fees, speaking fees, and/or honoraria from Rafa Laboratories, Syqe medical, Medison, Teva, Pfizer.
